# Left Gluteal and Perianal Eumycetoma Lesion Extending Into the Left Greater Sciatic Foramen, Pelvic Cavity to the External Anal Sphincter

**DOI:** 10.1002/ccr3.72316

**Published:** 2026-03-16

**Authors:** Rawa Badri, Ahmed Hassan Fahal

**Affiliations:** ^1^ The Mycetoma Research Centre University of Khartoum Khartoum Sudan

**Keywords:** eumycetoma, gluteal region, pelvic cavity, perianal area, sciatic foramen

## Abstract

Mycetoma is a chronic subcutaneous infection that primarily affects the lower extremities, although atypical anatomical presentations are possible. We describe a Sudanese farmer, age 31, who developed a left gluteal eumycetoma due to *Madurella mycetomatis*. Magnetic resonance imaging (MRI) showed widespread deep tissue involvement that extended into the pelvic cavity, sciatic foramen, and external anal sphincter despite a minor external lesion. Over the course of a year, the patient showed clinical improvement with lesion encapsulation while receiving oral itraconazole. In order to prevent advanced, debilitating complications, this case emphasizes the deceptive nature of mycetoma's superficial appearance, the vital role of MRI in disease assessment, and the significance of early detection through public health education in endemic regions.

## Key Clinical Message

The clinical manifestation of mycetoma can be misleading; minor skin lesions can mask deep tissue involvement. In cases with complex anatomy, magnetic resonance imaging is crucial for determining the extent of the disease. Presentation is frequently delayed by painless progression, social stigma, and low health literacy. To enhance early diagnosis, targeted awareness campaigns are required in endemic areas.

## 1 Introduction

Mycetoma presents as a persistent subcutaneous granulomatous inflammatory mass. It is caused by either true fungi or aerobic actinomycetes bacteria, and hence, it is classified as eumycetoma and actinomycetoma, respectively [1, 2]. It poses a significant health challenge, particularly in tropical and subtropical regions [3, 4]. The condition predominantly affects males, with a male‐to‐female ratio of 3.7:1, often attributed to increased exposure to soil organisms during outdoor activities [5, 6]. While mycetoma can occur at any age, it most commonly affects adults aged 20–40 years, who are typically the most socially active members of society, particularly in less developed countries [7]. The reported patient in this communication enjoyed all these characteristics.

The triad of a painless firm subcutaneous mass, multiple sinus formation, and a purulent or seropurulent discharge that contains grains is pathognomonic of mycetoma [8]. The foot is predominantly affected and is involved in 70% of patients [9]. Most of the lesions are present on the dorsal aspect of the forefoot. The hand is the next most common site, seen in 12% of patients, and the right hand is more often affected [9]. In endemic areas, other body sites may be involved but less frequently.

This case report hypothesizes that the clinical presentation of mycetoma significantly underestimates the true extent of disease, with superficial lesions masking substantial deep tissue involvement.

The objectives are to: (1) document an atypical anatomical presentation of eumycetoma with extensive invasion of the gluteal, perianal, and pelvic regions including the sciatic foramen and external anal sphincter; (2) demonstrate the diagnostic utility of advanced imaging modalities, particularly Magnetic resonance imaging (MRI), in delineating the full extent of subcutaneous and deep tissue pathology; (3) evaluate the clinical and therapeutic challenges associated with mycetoma involving anatomically complex and surgically inaccessible regions; and (4) analyze the contributory factors including disease chronicity, social stigmatization, inadequate health literacy, and socioeconomic barriers that lead to delayed healthcare‐seeking behavior and advanced disease presentation, thereby informing the need for targeted public health interventions in endemic areas.

## 2 Case History and Examination

In this communication, a 31‐year‐old male farmer from El Gazeria State who presented at the Mycetoma Research Centre in 2018 with left gluteal eumycetoma for 3 years is reported. His condition started as a small, painless left gluteal region swelling of gradual onset and gradually increased in size. Multiple sinuses discharging black grains appeared, and then he began to experience local pain that radiated to both lower limbs. The patient underwent a wide local excision after 2 years of his symptoms' progression and before his current presentation to the centre, but did not recall any further details regarding the surgery; he then started to have moderate‐severity pain that increased with walking and decreased with taking painkillers, which was worse in the early morning and the evening. He had multiple non‐specific symptoms that included anosmia, blurring of vision, and a daily throbbing headache. That later was of moderate severity, extending from the right temporal to the right occipital region, with no specific timing that is relieved with medications and sometimes without taking any relieving agents. All these non‐specific symptoms were not related to the mycetoma lesion.

One week before his present presentation, he had colicky abdominal pain and constipation. He then developed diarrhea after taking some laxative pills, and the colicky abdominal pain disappeared. He also developed a urinary tract infection that responded to medical treatment. Moreover, he had itching over the suprapubic region and the scrotum. His past medical history was not contributory to his condition. He is single and of moderate socioeconomic status. There is no similar condition in his family.

**FIGURE 1:**
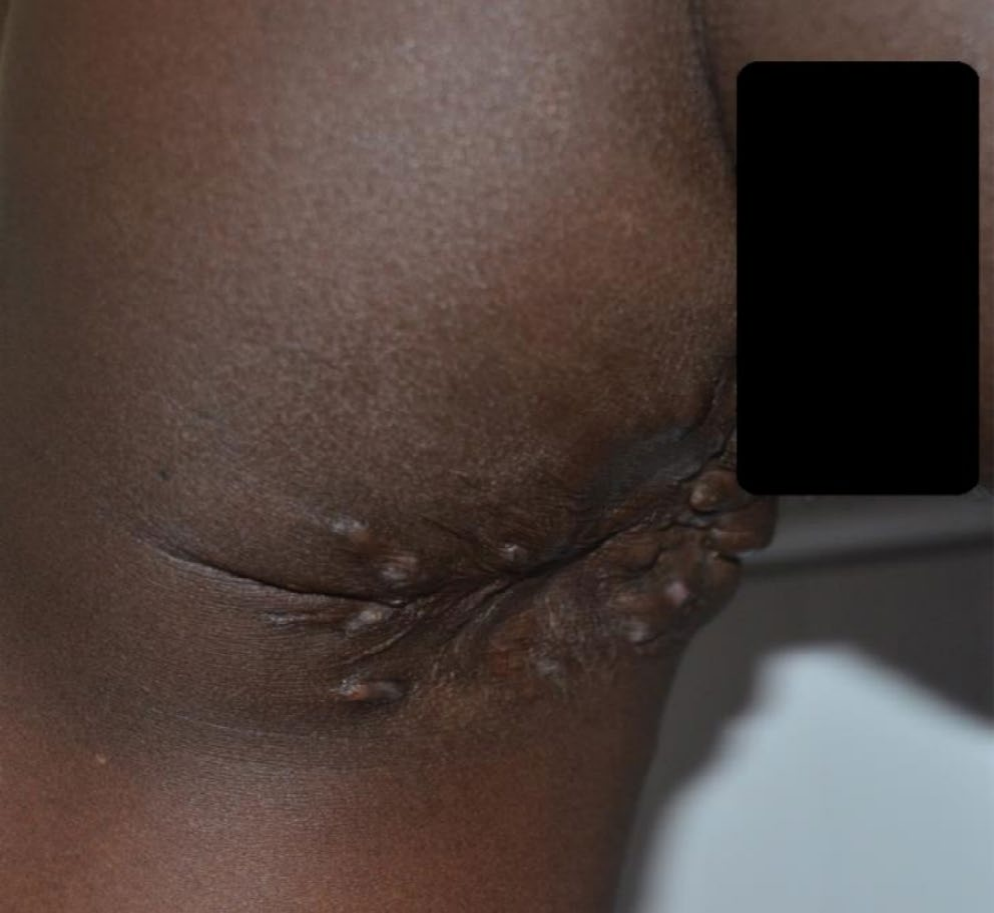
Photograph showing the perianal and left gluteal lesion with multiple sinuses.

On examination, the patient looked well, not pale, and was hemodynamically stable. The general systematic examination was within normal. The left gluteal region lesion was about 9 × 6 cm and irregular in shape. There were multiple small sinuses; some of them were active, discharging pus, but no grains were detected; the others were non‐active, and some were healed sinuses. The surgical scar from the previous wide local surgical excision was obvious. The lesion was neither tender nor of abnormal temperature (Figure 1).

Lower limb examination showed normal limb posture, no muscle wasting, fasciculation, or deformities. The lower limbs had a normal tone, power, reflexes, and sensations. The gait was normal.

## 3 Methods (Investigations, Differential Diagnosis, and Management)

**FIGURE 2:**
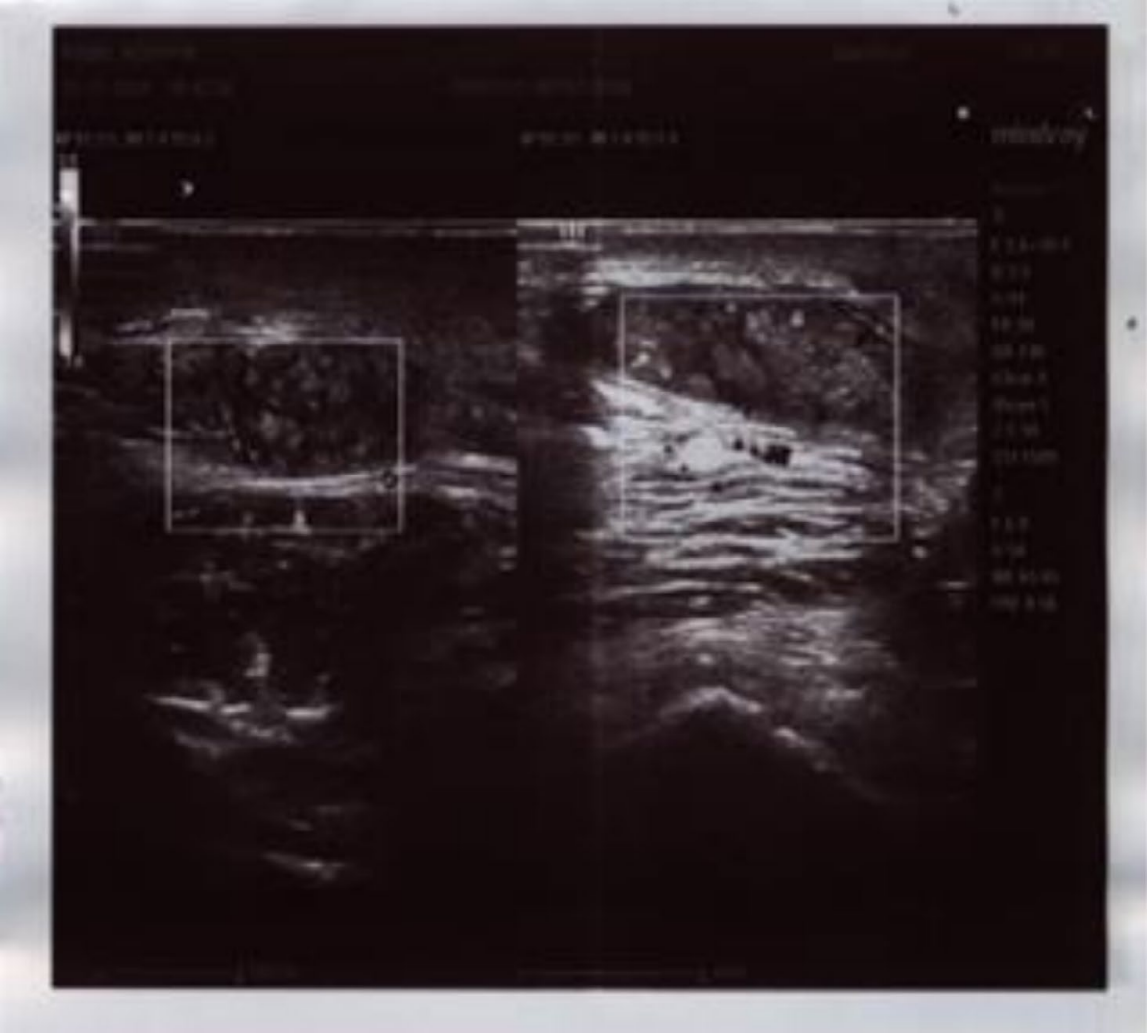
Ultrasound Scan of the lesion showing grains within pockets.

The patient's full blood count was within normal, apart from increased platelets of 848 × 10^3^/mm^3^. His random blood glucose, urine analysis, and renal and hepatic function tests were normal. Ultrasound scan showed a 3.2 × 2.5 × 1.6 cm left gluteal subcutaneous pocket containing grains extending through a 1 cm tract into an intermuscular location and connected to a multi‐lobulated 4.7 × 2.5 cm pocket at the perianal and left gluteus region (Figure 2).

**FIGURE 3:**
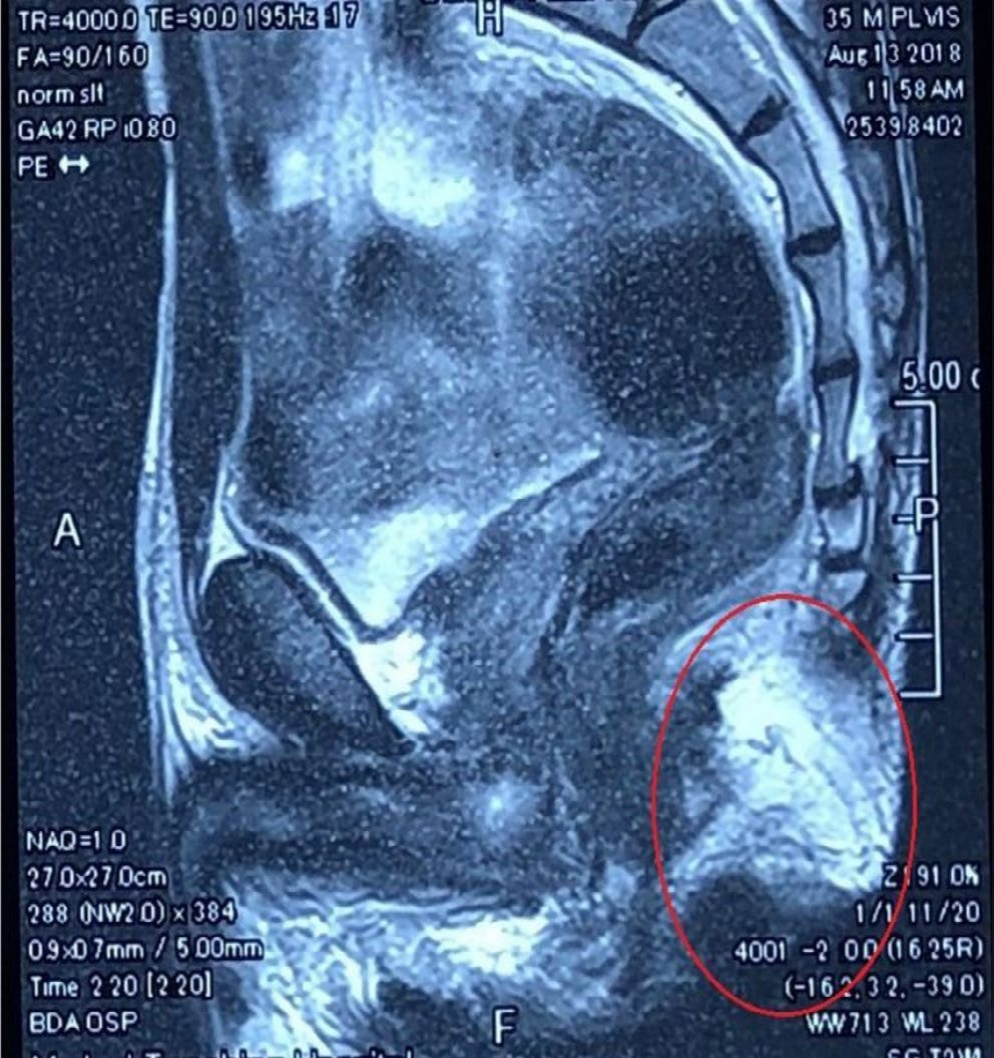
Magnetic resonance imaging (MRI) showing the lesion extending along the left ischio‐anal and ischio‐rectal fossae and reaching the left side of the anal canal and external anal sphincter.

**FIGURE 4:**
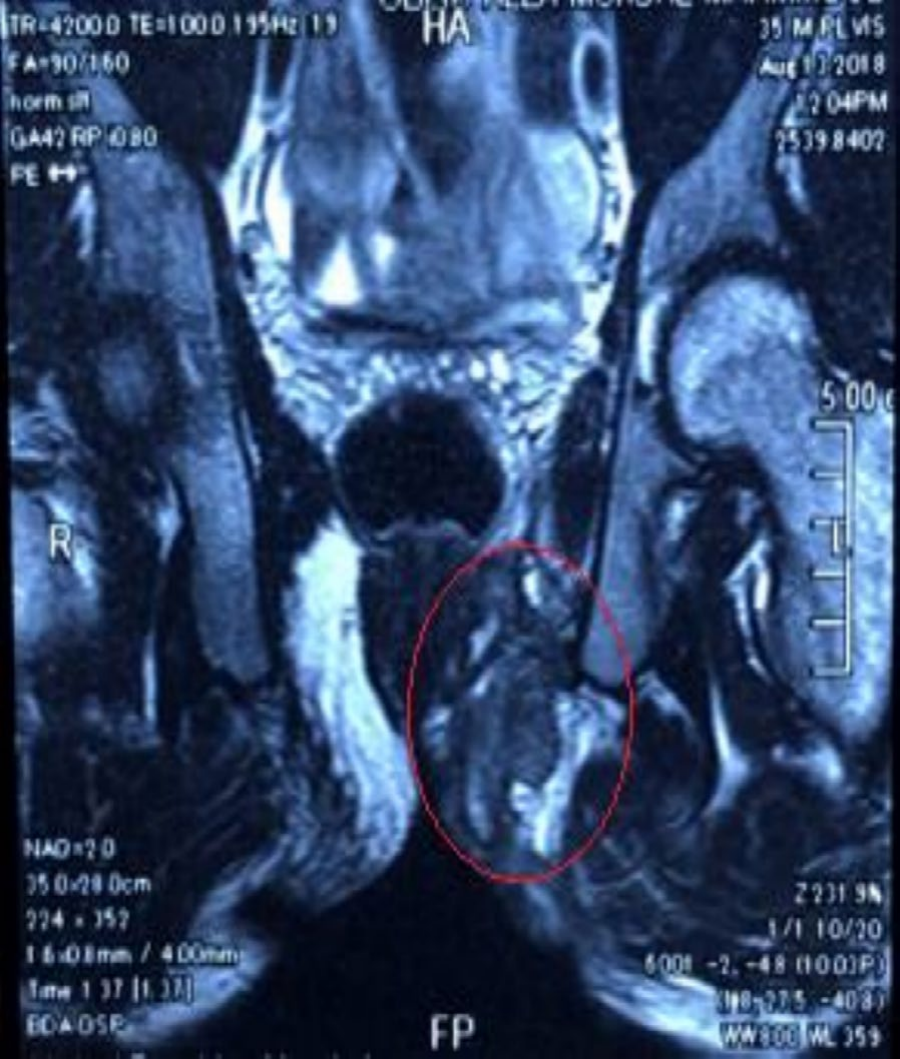
Magnetic resonance imaging (MRI) showing the lesion extending superiorly into the left levator plate, piercing the left posterolateral aspect of the anorectal junction and lower rectum.

**FIGURE 5:**
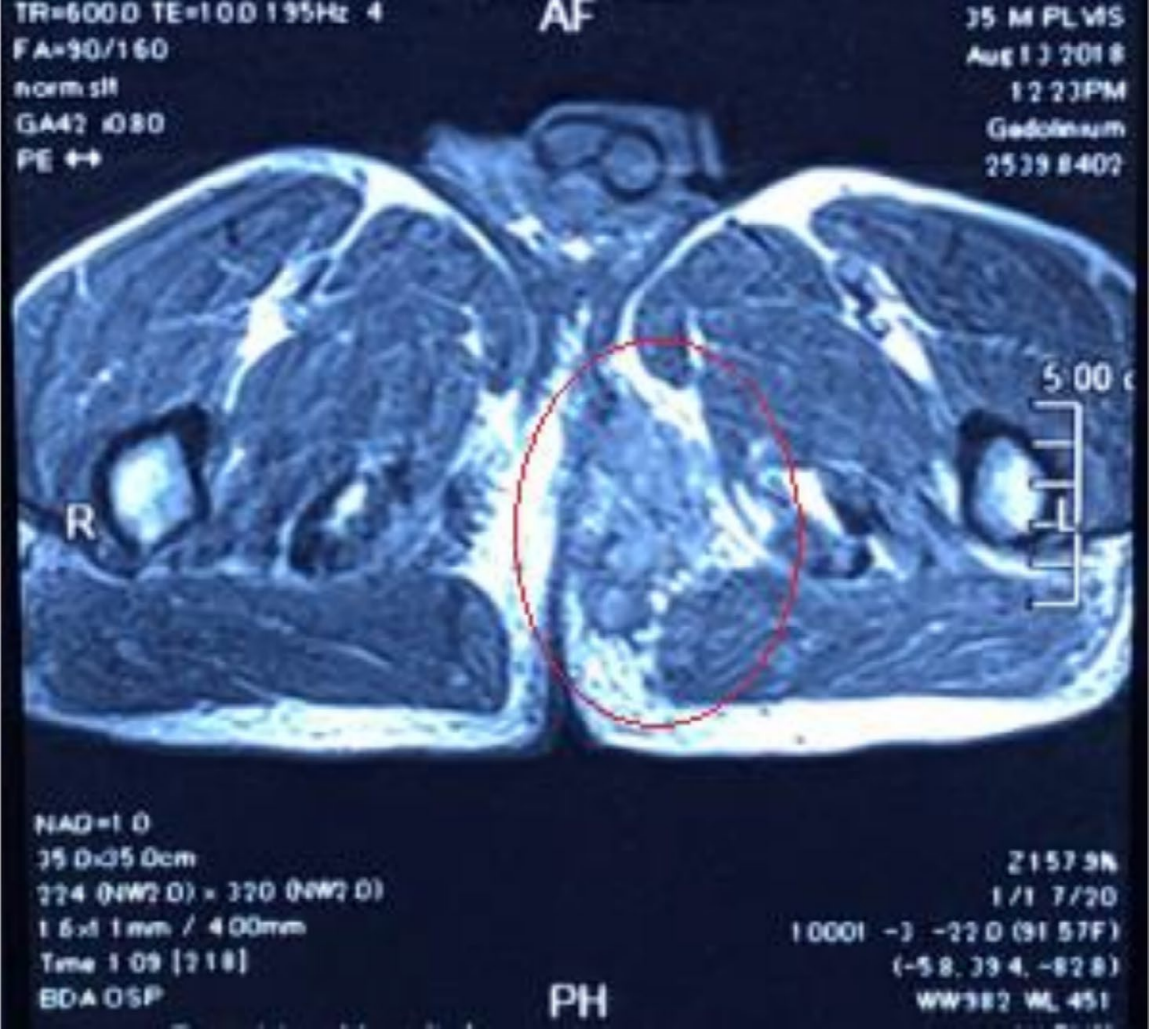
Magnetic resonance imaging (MRI) showing the lesion extending laterally deep to the left gluteus and piriformis muscles and into the left greater sciatic foramen with related intermuscular edema.

MRI showed fluid‐filled tracts and pockets at the left perianal and left gluteal region extending along the left ischio‐anal and ischio‐rectal fossae and reaching the left side of the anal canal and external anal sphincter (Figure 3). It was also extending superiorly into the left levator plate, piercing the left posterolateral aspect of the anorectal junction and lower rectum (Figure 4) and laterally deep to the left gluteus and piriformis muscles and into the left greater sciatic foramen with related intermuscular oedema (Figure 5).

**FIGURE 6:**
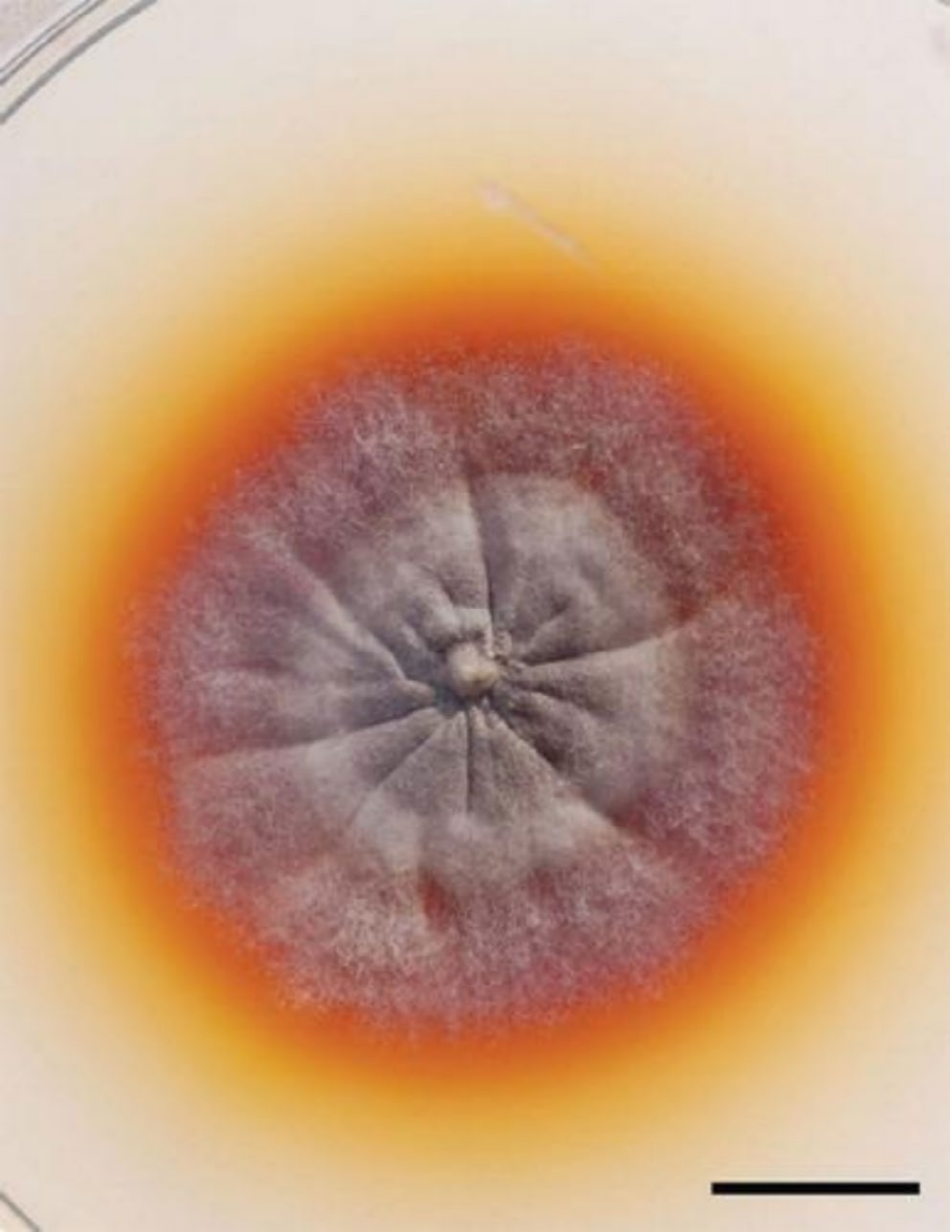
Showing the growth of *Madurella mycetomatis* colonies on Sabouraud dextrose agar [10].

Cytological examination of the lesion aspirates revealed *Madurella mycetomatis* grains. Grains from the lesion were collected and cultured on Sabouraud dextrose agar, and *M. mycetomatis* colonies were identified morphologically (Figure 6). The PCR confirmed the causative agent as *M. mycetomatis*.

## 4 Conclusion and Results

The patient was started on 200 mg oral itraconazole BD, Folic acid 0.5 mg OD daily, and Ibuprofen 400 mg when needed. He was on a 6‐week outpatient follow‐up. On the first outpatient follow‐up, all the abdominal problems and the lower limb pain had disappeared. On examination, the patient looked well but was pale. He was vitally stable with a slightly raised blood pressure of 152/105 mmHg. CBC and LFT were normal. Examination of the lesion revealed a hard and encapsulated lesion. The patient was advised to take a Dettol Sitz bath after each bowel movement and to continue medical treatment. The patient was referred to a physician due to his high blood pressure. After investigations, he was diagnosed with hypertension and started to take Amlodipine 5 mg OD daily and Aspirin 100 mg OD daily. The high blood pressure was an incidental finding and was not related to his lesion. One year later, the clinical examination of the patient revealed a well‐encapsulated, hard, small‐sized lesion of 4 × 3 cm. The patient was advised to continue on the same treatment plan and come back for the follow‐up, but the patient was lost for the follow‐up.

## 5 Case Discussion

A study documenting 20 cases of perianal mycetoma in Mexico associated its development with minor local injuries caused by soil, different vegetables, herbs, leaves, corn cobs, and branches used for anal hygiene post‐defecation [11]. These activities result in the subcutaneous introduction of the microorganisms responsible for the infection. However, in the reported patient, there was no history of local minor trauma.

The expansion of the lesion into the piriformis muscle may result in increased left buttock discomfort, particularly exacerbated by prolonged periods of sitting. The inflammatory granuloma progression into the puborectalis muscle and the left sciatic foramen could explain the right lower limb pain, as the sciatic nerve may be affected by surrounding tissue swelling.

Mycetoma commonly manifests as painless, which contributes significantly to delayed patient presentation. The majority of patients exhibit a prolonged duration of illness upon seeking medical attention, a constant finding in the medical literature. This delay is often linked to factors such as insufficient health education, patients' socioeconomic challenges, and the inadequacy of medical resources in endemic areas [12]. The reported patient is not an exception.

Mycetoma affecting delicate areas like the genitalia, perineum, scrotum, and gluteal regions is often perceived as socially stigmatizing. Consequently, patients frequently hesitate to seek medical help and delay treatment until the condition has progressed significantly [13].

Accurate diagnosis of mycetoma is essential for devising effective treatment strategies. Distinguishing between mycetoma and other lesions can be accomplished through the distinct ultrasonographic features. In eumycetoma cases, the lesions exhibit sharp, bright, hyper‐reflective echoes resembling black grains, setting them apart from actinomycetoma [14]. The presence of a “dot‐in‐circle” sign on MRI can offer a preliminary indication of the diagnosis [15]. Fine‐needle aspiration cytology (FNAC), cell blocks, and imprint cytology are the diagnostic techniques employed for mycetoma. Grain culture and histopathological and molecular techniques can settle the diagnosis of mycetoma [16].

Mycetoma is a deep mycosis, and the external appearance is always deceiving; it represents the tip of the iceberg. The reported patient presented with a small gluteal lesion that had widely spread in the deep pelvic cavity structures. Determining the disease extension is essential to plan proper management. The lesional MRI examination revealed the deep‐seated inflammatory granuloma spread. However, the MRI facility is only available in a few centers in the endemic regions.

Surgical intervention in these areas poses challenges due to their intricate anatomy, and achieving complete excision is often difficult, leading to a high rate of recurrence [17].

Additionally, the proximity of the anal canal increases the risk of secondary bacterial infection, complicating the affected area's anatomy and ultimately leading to unfavorable treatment outcomes [18]. Under such circumstances, it is recommended to persist with mycetoma medical therapy while managing the secondary bacterial infection. The objective is to improve lesion localisation and encapsulation, making them more suitable for surgical removal.

The painless nature of mycetoma, coupled with insufficient health education, led to the reported patient's late presentation for treatment. Hence, it is advisable to conduct educational campaigns aimed at increasing awareness of mycetoma in endemic regions. This initiative aims to facilitate early detection and treatment, thereby averting the onset of severe and debilitating complications.

## Author Contributions

**Rawa Badri:** conceptualization, writing – original draft, writing – review and editing. **Ahmed Hassan Fahal:** supervision, writing – review and editing.

## Funding

The authors have nothing to report.

## Consent

Written informed consent was obtained from the patient to publish this report in accordance with the journal's patient consent policy.

## Conflicts of Interest

The authors declare no conflicts of interest.

## Data Availability

Data sharing not applicable to this article as no datasets were generated or analyzed during the current study.
